# Integrated Cross-Platform Analysis Reveals Candidate Variants and Linkage Disequilibrium-Defined Loci Associated with Osteoporosis in Korean Postmenopausal Women

**DOI:** 10.3390/diagnostics16010153

**Published:** 2026-01-03

**Authors:** Su Kang Kim, Seoung-Jin Hong, Seung Il Song, Jeong Keun Lee, Gyutae Kim, Byung-Joon Choi, Suyun Seon, Seung Jun Kim, Ju Yeon Ban, Sang Wook Kang

**Affiliations:** 1 Department of Biomedical Laboratory Science, Catholic Kwandong University, Gangneung 25601, Republic of Korea; skkim7@cku.ac.kr; 2 Department of Prosthodontics, College of Dentistry, Kyung Hee University, Seoul 02447, Republic of Korea; ssabock@khu.ac.kr; 3 Department of Dentistry, School of Medicine, Ajou University, Suwon 16499, Republic of Korea; ssi1219@ajou.ac.kr (S.I.S.); arcady@ajou.ac.kr (J.K.L.); 4 Department of Oral and Maxillofacial Radiology, College of Dentistry, Kyung Hee University, Seoul 02447, Republic of Korea; latinum.omfr@khu.ac.kr; 5 Department of Oral and Maxillofacial Surgery, College of Dentistry, Kyung Hee University, Seoul 02447, Republic of Korea; sjnb2@khu.ac.kr; 6 Department of Dentistry, Graduate School, Kyung Hee University, Seoul 02447, Republic of Korea; hillario84@naver.com (S.S.); june6month@hanmail.net (S.J.K.); 7 Department of Dental Pharmacology, College of Dentistry, Dankook University, Cheonan 31116, Republic of Korea; jyban@dankook.ac.kr; 8 Department of Oral and Maxillofacial Pathology, College of Dentistry, Kyung Hee University, Seoul 02447, Republic of Korea

**Keywords:** osteoporosis, Illumina Infinium HumanExome BeadChip, Affymetrix Axiom Exome Array, linkage disequilibrium analysis, machine learning, postmenopausal women, Korean genome and epidemiology study

## Abstract

**Background:** Osteoporosis is highly prevalent in postmenopausal women, yet genome-wide association studies often miss disease-relevant variants because of incomplete single nucleotide polymorphism (SNP) coverage and platform-specific limitations. We aimed to identify genetic contributors to osteoporosis risk by integrating two exome-based genotyping platforms with multilayer analytic approaches. **Methods:** We analyzed extreme osteoporosis phenotypes in Korean postmenopausal women from the Korean Genome and Epidemiology Study (KoGES) Ansan–Anseong cohorts using the Illumina Infinium HumanExome BeadChip and the Affymetrix Axiom Exome Array. After standard quality control, single-SNP logistic regression, cross-platform overlap analysis, and three machine-learning models were applied. Predicted functional impact was evaluated using multiple in silico algorithms and conservation scores. Finally, datasets from both platforms were merged, and cross-platform linkage disequilibrium (LD) blocks were defined to identify loci containing SNPs with *p* < 1 × 10^−4^. **Results:** No overlapped SNP reached genome-wide significance, but rs2076212 in *PNPLA3* achieved suggestive significance (*p* < 1 × 10^−5^) only on the Illumina array. Cross-platform analysis identified 111 overlapping SNPs in 70 genes. Integrated machine-learning, in silico, and conservation evidence prioritized *ARMS2*, *CCDC92*, *NQO1*, *ZNF510*, *PTPRB*, and *DYNC2H1* as candidate genes. LD-block analysis revealed 10 blocks with at least one SNP at *p* < 1 × 10^−4^, including four chromosome 12 loci (*NAV2*, *BICD1*, *CCDC92*, *ZNF664*) that became apparent only when LD patterns were evaluated jointly across platforms. **Conclusions:** Combining dual exome arrays with LD-block analysis, machine learning, and functional prediction improved sensitivity for detecting low bone mineral density-related loci and highlighted *CCDC92*, *DYNC2H1*, *NQO1*, and related genes as biologically plausible candidates for future validation.

## 1. Introduction

Osteoporosis is a disease characterized by weakened bones that become easily fractured due to low bone mineral density (BMD) and microstructural changes. It is particularly prevalent among postmenopausal women and the older adults. The World Health Organization (WHO) defines osteoporosis as a condition where bone mineral density is −2.5 or lower when comparing bone density measurements to the average bone density of young adults [1]. Osteoporosis is a widespread health issue globally, affecting over 200 million people aged 50 and older. According to the Korea Disease Control and Prevention Agency, 37.3% of women and 7.5% of men over the age of 50 in South Korea have been diagnosed with osteoporosis (www.kdca.go.kr). The prevalence of osteoporosis increases rapidly with age, affecting approximately two-thirds of women and one-fifth of men aged 70 and older.

Despite being a common condition, osteoporosis can have severe, even fatal, consequences. The most significant risk associated with osteoporosis is fractures, which can lead to serious complications. Hip fractures, in particular, often result in mobility loss and prolonged bed rest, increasing the risk of complications such as bedsores and pneumonia. With a mortality rate of approximately 20% within one year following a hip fracture, osteoporosis should therefore be regarded as a serious health threat [2].

The development of osteoporosis is influenced by a wide range of factors, including age, menopause, lifestyle habits such as smoking, alcohol consumption, and physical inactivity, as well as nutritional deficiencies and the use of certain medications [3]. Among these risk factors, genetic predisposition has been increasingly recognized as a major contributor. Numerous studies have shown that genetic influences substantially affect both the onset of osteoporosis and the reduction in bone mineral density. Twin studies, in particular, have indicated that up to 60–80% of the variance in bone density can be attributed to genetic factors, underscoring the importance of genetic predisposition in the pathogenesis of osteoporosis risk or low BMD [4]. To date, most genetic research on osteoporosis risk or low BMD has been conducted using genome-wide association studies (GWAS), which are widely used to identify genetic variants associated with complex diseases [5]. Previous GWAS investigating bone mineral density (BMD) in Korean populations have mainly focused on DXA-based phenotypes and used genotyping or multi-ethnic meta-analyses to improve genome coverage, but have typically relied on single genotyping platforms [6,7]. However, incomplete SNP coverage, reduced power to detect rare variants, and the limited coverage of exonic variants and the dependence on a single platform can lead to missed trait-relevant loci [8].

To address these limitations, this study employed two complementary genotyping platforms: the Illumina Exome Chip and the Affymetrix Axiom Exome Array. Using both arrays expands SNP coverage through distinct probe sets, enhances the detection of rare variants, enables cross-validation of significant loci to reduce false positives and negatives, and mitigates platform-specific biases, thereby improving the reliability and reproducibility of GWAS results. The Illumina Exome Chip and the Affymetrix Axiom Exome Array, commonly used platforms for genetic analysis, each possess distinct advantages and limitations. The Illumina Exome Chip is tailored to rare coding variants such as missense and nonsense mutations, but its design—based primarily on European-ancestry exomes—limits representation in non-European populations, and array-based genotyping constraints result in ~20–30% of targeted variants failing probe design or accurate genotyping. Moreover, statistical power for detecting low-frequency and especially ultra-rare variants remains limited [9,10,11]. In contrast, the Affymetrix Axiom Exome Array exhibits high accuracy and reproducibility across diverse variants, yet shows a marked decline in positive predictive value for ultra-rare heterozygous calls, leading to false positives [12,13].

While analyzing two genotyping platforms simultaneously is highly valuable, single-SNP association results can appear inconsistent—even when reflecting the same underlying biological signal—due to differences in variant density, probe design, and tagging efficiency. To address these limitations, this study incorporated LD block–level analysis to group correlated SNPs into coherent genomic loci [14,15]. This approach enables key SNPs from both platforms to be integrated within the same LD-defined regions, thereby improving the robustness of cross-platform validation, particularly for rare or poorly tagged variants.

Therefore, the aim of this study is to simultaneously analyze data obtained from the Illumina Infinium HumanExome BeadChip and the Affymetrix Axiom Exome Array and to cross-validate the findings from each platform in order to more accurately identify genes associated with low BMD and osteoporosis risk. The ultimate goal is to elucidate the genetic underpinnings of low BMD and osteoporosis risk, thereby providing foundational insights that can inform future efforts in disease prediction, diagnosis, and the development of personalized treatment strategies.

## 2. Materials and Methods

### 2.1. Study Subjects

**Figure 1:**
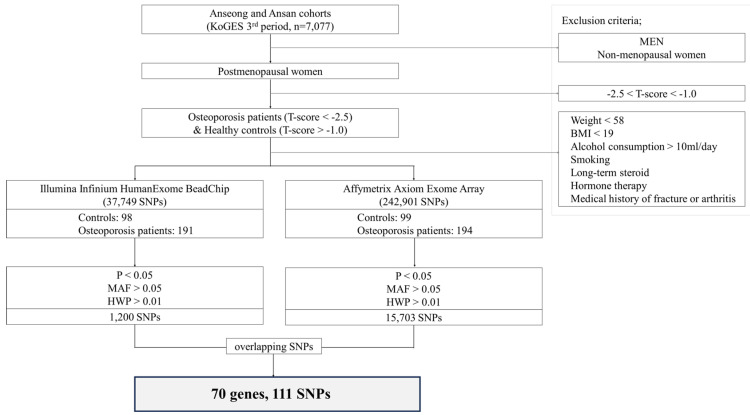
Flowchart of participant selection and SNP filtering process. Flowchart summarizing the selection of postmenopausal women from the Ansan and Anseong cohorts of the KoGES, 3rd period, and the subsequent SNP filtering steps. After applying exclusion criteria, participants were genotyped using two independent platforms (Illumina Infinium HumanExome BeadChip and Affymetrix Axiom Exome Array). SNPs were filtered by statistical significance, allele frequency, and HWE, and overlapping variants were identified for further analysis.

This study was conducted using data from the third phase of the Anseong and Ansan cohorts of the Korean Genome and Epidemiology Study (KoGES), which included a total of 7077 subjects. Figure 1 presents a flowchart outlining the subject selection and analysis process. Initially, men and premenopausal women were excluded, and only postmenopausal women were included in the analysis. In this study, we used bone status indices based on Sunlight OmniSense quantitative ultrasound (QUS) measurements to define osteoporosis risk. Previous studies have reported that QUS measurements are significantly correlated with DXA measurements, particularly in postmenopausal women [16]. T- and Z-scores were derived from speed-of-sound (SOS) measurements [17]. Specifically, low BMD was defined as participants with a T-score < −2.5 and a Z-score < −2.0, as measured by QUS at the midshaft tibia or distal radius. This operational definition was used to maximize phenotypic contrast and differs from the WHO/ISCD clinical diagnostic criteria [1], which rely on central DXA measurements at the lumbar spine or hip. Healthy controls were defined as participants with a QUS-derived T-score ≥ −1.0, and to further enhance separation between cases and controls, participants with T-scores between −2.5 and −1.0 were excluded from the analysis. To isolate the genetic influence on low BMD, subjects with known risk factors such as body weight < 58 kg, body mass index (BMI) < 19, alcohol consumption > 10 mL/day, smoking, long-term steroid use, hormone therapy, and a medical history of fracture or arthritis were also excluded [1,18].

The final selected participants were as follows:

• Illumina Infinium HumanExome BeadChip (Illumina, Inc., San Diego, CA, USA) group: 98 healthy controls and 191 low BMD cases (total *n* = 289).
• Affymetrix Axiom Exome Array group: 99 healthy controls and 194 low BMD cases (total *n* = 293).

From the Illumina Infinium HumanExome BeadChip, 30,538 single nucleotide polymorphisms were analyzed, and a total of 1200 SNPs met the quality control (QC) thresholds of *p* < 0.05, minor allele frequency (MAF) > 0.05, and Hardy–Weinberg equilibrium (HWE) > 0.01, which were applied to minimize potential false-positive associations [19]. From the Affymetrix Axiom Exome Array (Thermo Fisher Scientific Inc., Waltham, MA, USA), 242,901 SNPs were examined, and 15,703 SNPs met the same criteria. A cross-platform comparison revealed that 111 SNPs across 70 genes were commonly identified as statistically significant in both datasets. This study was approved by the Institutional Review Board of Dankook University (IRB No. 2018-08-004 Date: 10 September 2018). As indicated in the IRB documents, this study was classified as a retrospective cohort/genetic data–based analysis, and therefore written informed consent was waived. The IRB approved this exemption and included a “Written Consent Waiver Statement” in the official documentation. Accordingly, no participant consent form was required, and none was collected.

### 2.2. Statistical Analysis

Group comparisons between low BMD cases and healthy controls were carried out using independent *t*-tests implemented in Python 3.11.13. To identify genetic variants potentially linked to low BMD, logistic regression models were constructed using both SNP & Variation Suite (Golden Helix, Bozeman, MT, USA) and Persistent Linked INtegrated Kit version 1.9 (PLINK) [20]. Visualization of genome-wide association signals was performed through Manhattan and quantile-quantile (Q-Q) plots using Python 3.11.13. The chromosomal positions of significant SNPs were mapped using the PhenoGram visualization tool (http://visualization.ritchielab.org [accessed on 02 October 2018]) [21]. To reduce model-specific bias and enhance the confidence of common features, three models—linear discriminant analysis (LDA), random forest, and XGBoost model—were used to capture both linear (additive) effects and non-linear SNP interactions, thereby providing complementary information for variant prioritization. Machine learning analyses were applied as an auxiliary and exploratory approach to prioritize candidate SNP features associated with case status (OP = 1) versus controls (OP = 0). Linear discriminant analysis (LDA) was used to derive a one-dimensional discriminant component and to rank SNPs based on the absolute values of discriminant coefficients. Random forest and XGBoost classifiers were trained using an 80/20 train–test split, and SNPs were ranked by model-based feature importance scores. The top-ranked SNPs (top 10) from each model were visualized using PCA (tree-based models) and LDA projection (LDA), to illustrate separation patterns. To assess the potential functional impact of non-synonymous variants, we obtained functional prediction scores using the dbNSFP database [22], including the Sorting Intolerant from Tolerant (SIFT), Polymorphism Phenotyping v2, HumDiv-trained model/HumVar-trained model (PolyPhen-2 HDIV/HVAR), Protein Variation Effect Analyzer (PROVEAN), Rare Exome Variant Ensemble Learner (REVEL), and Combined Annotation-Dependent Depletion (CADD). Thresholds for predicting deleteriousness were set as follows: SIFT score < 0.05 [23], PolyPhen-2 HDIV/HVAR score > 0.85 for “probably damaging” [24], PROVEAN score < –2.5 [25], REVEL score > 0.5 [26], and CADD phred ≥ 20 [27]. To explore the evolutionary conservation of the overlapping SNPs, conservation scores were obtained using Genomic Evolutionary Rate Profiling (GERP++) [28], Phylogenetic *p*-value (phyloP) [29], and Phylogenetic Hidden Markov Model Conservation Score (phastCons) [30]. To explore protein–protein interaction networks, we used the Search Tool for the Retrieval of Interacting Genes/Proteins (STRING) v12.0 with a minimum required interaction score of 0.400, and clustering was performed using Markov Cluster Algorithm (MCL) clustering (inflation parameter = 2.0) (https://string-db.org [accessed on 16 August 2025]) [31]. Functional enrichment and pathway analysis of the identified genes were performed using Database for Annotation, Visualization, and Integrated Discovery (DAVID; https://davidbioinformatics.nih.gov [accessed on 16 August 2025]) [32]. Post hoc power analysis was conducted for selected SNPs highlighted in the main association results to evaluate whether the available sample size was sufficient to detect the observed effect sizes under a predefined genome-wide significance threshold (α = 1 × 10^−5^), using Python (version 3.11.13). Selection of SNPs was based on observed effect sizes (OR > 1.3 or < 0.7) and minor allele frequency (MAF) > 0.05. Minimum detectable odds ratios at 80% power were calculated using a Wald test–based normal approximation under an additive logistic regression model, incorporating sample size, case–control ratio, and minor allele frequency.

### 2.3. Linkage Disequilibrium Block Analysis and SNP Characterization

Genotype data obtained from the Illumina HumanExome BeadChip and the Affymetrix Axiom Exome Array were combined into a single working dataset. Before merging, both datasets underwent standard quality control using PLINK (version 1.9), including filters for call rate, minor allele frequency, and Hardy–Weinberg equilibrium. Because the two platforms use slightly different probe designs and allele encodings, we harmonized allele formats (including strand alignment) and updated SNP identifiers and genomic coordinates to ensure that variants aligned correctly across platforms. After preprocessing, pairwise linkage disequilibrium (LD) values (r^2^) were calculated using PLINK. LD blocks were then defined using a graph-based approach in which SNPs were treated as nodes and pairwise LD relationships (r^2^ ≥ 0.8) were represented as edges. LD blocks were identified as connected components within this SNP network, and SNP-to-block assignments were recorded for downstream block-level analyses. To focus on regions with potential biological relevance, we retained only LD blocks that included at least one SNP with an association *p*-value below 1 × 10^−4^. Within each selected block, the SNP with the smallest *p*-value was designated as the lead variant. For visualization and quality assessment, LD heatmaps were generated in R (version 4.3.3) by constructing block-wise r^2^ matrices, ordering SNPs by physical position, and visualizing within-block LD structure. This approach allowed inspection of LD consistency and block integrity across the two genotyping platforms. For each block, we also generated a LocusZoom-style plot to visualize the local association pattern around the lead SNP.

## 3. Results

### 3.1. Characteristics of Study Subjects

Age was significantly higher in the low BMD group, although age at menopause showed no significant difference. Alcohol and calcium intake were also comparable between groups. Notably, both body weight and BMI were significantly higher in the low BMD group. Moreover, while distal radius speed of sound (DR-SOS), distal radius T-score (DR-T), and distal radius Z-score (DR-Z) did not differ between groups, midshaft tibia speed of sound (MT-SOS), midshaft tibia T-score (MT-T), and midshaft tibia Z-score (MT-Z) demonstrated significant differences (Table 1).

### 3.2. Q-Q Plots and Manhattan Plots of Logistic Regression

**Figure 2:**
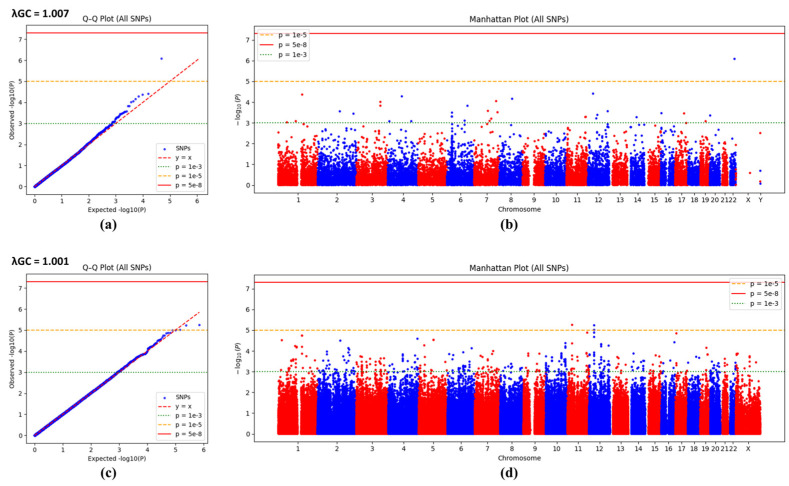
Q-Q plots and Manhattan plots of logistic regression *p*-values for low BMD (cases vs. controls). (**a**) Illumina Infinium HumanExome BeadChip Q–Q plot, (**b**) Illumina Infinium HumanExome BeadChip Manhattan plot, (**c**) Affymetrix Axiom Exome Array Q-Q plot, and (**d**) Affymetrix Axiom Exome Array Manhattan plot. Dots represent the –log_10_(*p*) values of SNPs, alternately colored blue and red for each chromosome. Horizontal lines indicate *p* = 1 × 10^−3^ (green dotted line), *p* = 1 × 10^−5^ (orange dotted line), and *p* = 5 × 10^−8^ (red solid line; genome-wide cutoff). No SNP exceeds the genome-wide cutoff in any panel. In the Illumina exome plot (**b**), some SNPs exceed the suggestive threshold (*p* < 1 × 10^−5^), whereas in the Affymetrix plot (**d**), the signals are generally weaker. Q-Q plots are largely consistent with the expected line (y = x) except for deviations in the tails, suggesting that systematic bias is minimal. Blue and red dots in (**b**,**d**) indicate alternating chromosomes for visual distinction only.

Figure 2 shows the Q-Q plots and Manhattan plots from genome-wide association analyses using the Illumina Infinium HumanExome BeadChip and the Affymetrix Axiom Exome Array. Neither platform identified any SNPs meeting the genome-wide significance threshold (*p* < 5 × 10^−8^). In the Illumina Infinium HumanExome BeadChip data, one SNP exceeded the suggestive significance threshold (*p* < 1 × 10^−5^), and several SNPs displayed slight deviations from the expected line in the upper tail below the suggestive threshold, although the overall distribution closely matched the expected line. In the Affymetrix Axiom Exome Array data, association signals were generally lower, with few SNPs approaching or reaching the suggestive threshold, and distinct clusters were limited. The genomic inflation factors indicated minimal inflation for both platforms (λGC = 1.007 and 1.001, respectively).

### 3.3. Overlapping SNPs Across Two Genotyping Platforms (111 SNPs Across 70 Genes)

**Figure 3:**
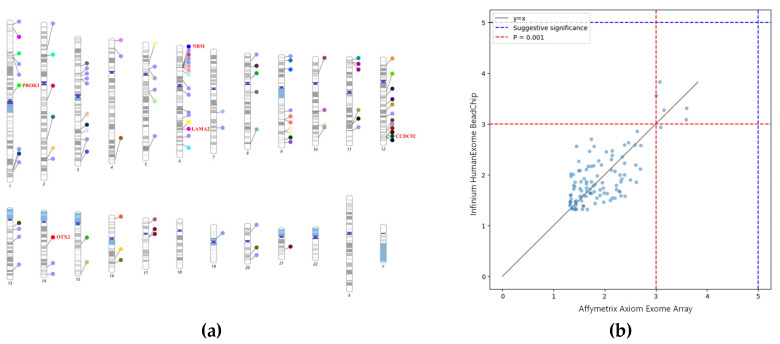
Overlapping variants identified across two genotyping platforms. (**a**) Chromosomal ideogram showing the genomic locations of genes harboring overlapping SNPs detected in both the Illumina Infinium HumanExome BeadChip and the Affymetrix Axiom Exome Array. Each colored circle represents a unique gene, and red-labeled genes indicate those with the lowest *p*-values. (**b**) Scatter plot comparing –log10(*p*) values for overlapping SNPs between the two platforms. Red dashed lines indicate a nominal significance level (*p* = 0.001), and the blue dashed line marks the suggestive significance threshold (*p* = 1 × 10^−5^). No SNP reached the genome-wide significance threshold (*p* < 5 × 10^−8^); however, rs2076212 exhibited suggestive significance on the Illumina platform. The different dot colors in Figure 3a are used solely to visually distinguish SNP loci.

Figure 3a presents a chromosome map showing the locations of genes in which SNPs were commonly detected across both platforms, with each dot representing a distinct gene. Genes with the lowest *p*-values are highlighted in red. Table 2 lists the number of overlapping SNPs and their corresponding genes for each chromosome. Genes without overlapping SNPs were excluded. Notably, higher numbers of overlapping SNPs and genes were observed on chromosomes 1, 3, 6, 9, 11, and 12.

Figure 3b shows a scatter plot comparing the *p*-values (−log_10_ transformed) of SNPs analyzed from the same samples on the Illumina Infinium HumanExome BeadChip and the Affymetrix Axiom Exome Array. The red and blue dotted lines represent significance thresholds at *p* = 0.001 and *p* = 1 × 10^−5^, respectively. No SNPs reached the genome-wide significance threshold (*p* < 5 × 10^−8^) or the suggestive threshold (*p* < 1 × 10^−5^) on either platform.

### 3.4. Top SNP Selection via Multiple Machine Learning Models

In this study, we applied three machine learning techniques—LDA, random forest, and XGBoost—to assess the impact of SNPs with shared significance across two genome analysis platforms (Illumina Infinium HumanExome BeadChip and Affymetrix Axiom Exome Array) on osteoporosis risk. Table 3 presents the top 10 SNPs identified by each model for both platforms. SNPs with the highest coefficients or importance scores are listed for each model, enabling the identification of genes and SNPs common to multiple platforms and models. LDA results showed a relatively similar top SNP composition between platforms, whereas random forest and XGBoost yielded more divergent top SNP lists. Blank entries in the table indicate SNPs not directly linked to protein-coding genes.

### 3.5. Predicted Deleterious Non-Synonymous SNPs Identified Across Both Platforms by Multiple in Silico Algorithms

We analyzed overlapping SNPs identified across both platforms to identify SNPs with potential functional impact on proteins. Table 4 shows variants predicted to have deleterious or damaging effects by at least three of the following five algorithms (SIFT, PolyPhen2 HDIV/HVAR, PROVEAN, REVEL, and CADD). The prioritized SNPs were located in genes associated with including metabolic, cytoskeletal, and signal-transduction related genes, including *ARMS2*, *CCDC92*, *NQO1*, *ZNF510*, *PTPRB*, and *DYNC2H1*. Notably, *CCDC92*, *NQO1*, *ZNF510*, *PTPRB* and *DYNC2H1* showed CADD phred scores ≥ 20, suggesting that these variants are among the top 1–5% of deleterious substitutions in the human genome. Several mutations, such as *ARMS2* p.Ala69Ser and *DYNC2H1* p.Arg2871Pro, were consistently predicted to be damaging by four or more algorithms.

### 3.6. Conservation Analysis

We performed a conservation analysis of overlapping SNPs identified across both platforms using GERP++, phyloP, and phastCons scores to evaluate their evolutionary conservation. As shown in Table 5, several SNPs, including *DYNC2H1* (rs589623), *NQO1* (rs1800566), and *ZNF510* (rs2289651), exhibited high GERP++ scores (>2.0), indicating strong evolutionary constraint. PhyloP scores (both phyloP1 and phyloP4) were also markedly elevated for *NQO1* (9.295 and 8.644, respectively) and *DYNC2H1* (4.414 and 0.676), suggesting that these variants occur at highly conserved genomic positions across multiple species. Similarly, phastCons scores for *DYNC2H1* and *NQO1* approached 1.0, indicating strong probability of being in a conserved element.

### 3.7. Post Hoc Power and Minimum Detectable Effect Size Analysis

Post hoc power analysis was conducted to assess whether the sample size used in the SNP association analysis was sufficient to detect the observed effect sizes. Under a suggestive significance threshold (α = 1 × 10^−5^), most overlapping SNPs showed post hoc power values exceeding the predefined threshold. However, rs2584021 (*PTPRB*) exhibited low post hoc power (<0.20), suggesting limited statistical power for these variants. Among the non-redundant SNPs, rs2076212 (*PNPLA3*) was the only variant showing suggestive significance; however, its post hoc power was low (0.16), indicating that the observed association should be interpreted with caution due to the limited sample size. Nevertheless, although several SNPs showed statistically significant associations, none exceeded the minimum detectable odds ratio required for 80% power, indicating that the study remains underpowered to reliably detect small-to-moderate effect sizes given the modest sample size.

### 3.8. Protein–Protein Interactions and Functional Enrichment Analysis

**Figure 4:**
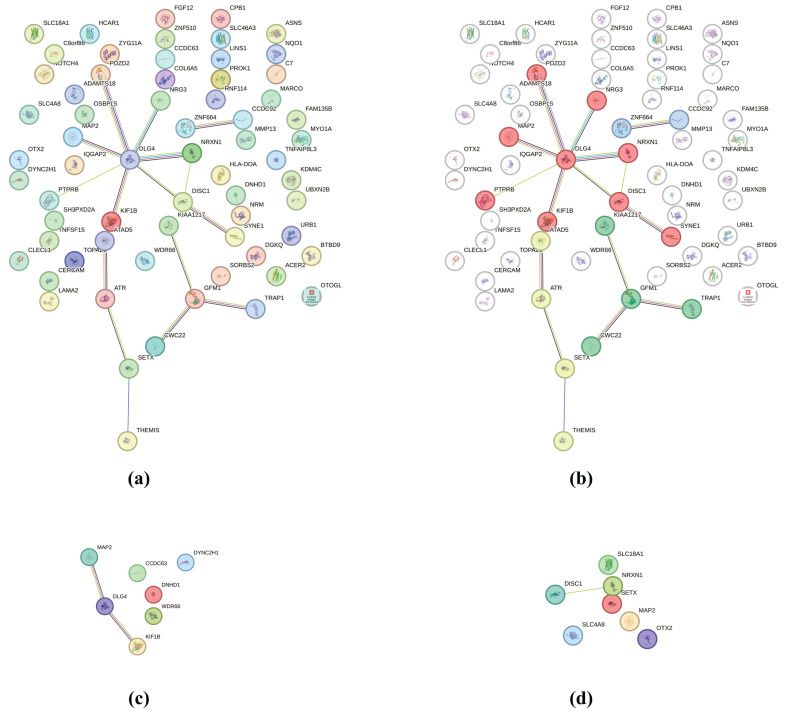
Protein–protein interaction (PPI) network and functional clustering of overlapping genes identified on both platforms. (**a**) STRING PPI network, (**b**) MCL-based clustered network showing functional grouping of genes (inflation = 2.0), (**c**) Subnetwork enriched for plasma membrane-bounded cell projection cytoplasm (FDR = 9.41 × 10^−11^), (**d**) Subnetwork enriched for distal axon (GO:0150034; FDR = 2.71 × 10^−10^). Nodes represent proteins encoded by overlapping genes, and colors indicate cluster membership.

We performed protein–protein interaction (PPI) analysis using the overlapping genes identified on both platforms. As shown in Figure 4a, the STRING network consisted of 65 nodes and 16 edges, with an average node degree of 0.492 and an average local clustering coefficient of 0.221. The PPI enrichment *p*-value was 0.122, indicating that the network did not contain significantly more interactions than would be expected by chance. Although not statistically enriched, several nodes showed multiple connections, which may imply limited functional grouping (Table 6). After MCL clustering, Figure 4b revealed functionally grouped modules. The largest cluster was enriched for genes involved in cytoskeletal organization and cellular projection–related processes. To further explore functional enrichment, subsets of genes were analyzed using STRING Gene Ontology categories. Figure 4c shows a cluster enriched for plasma membrane-bounded cell projection cytoplasm (GO:0032838; FDR = 9.41 × 10^−11^), including several microtubule-associated genes. Figure 4d shows another cluster related to distal axon (GO:0150034; FDR = 2.71 × 10^−10^).

### 3.9. LD Block Analysis and Cross-Platform Locus Characterization

**Figure 5:**
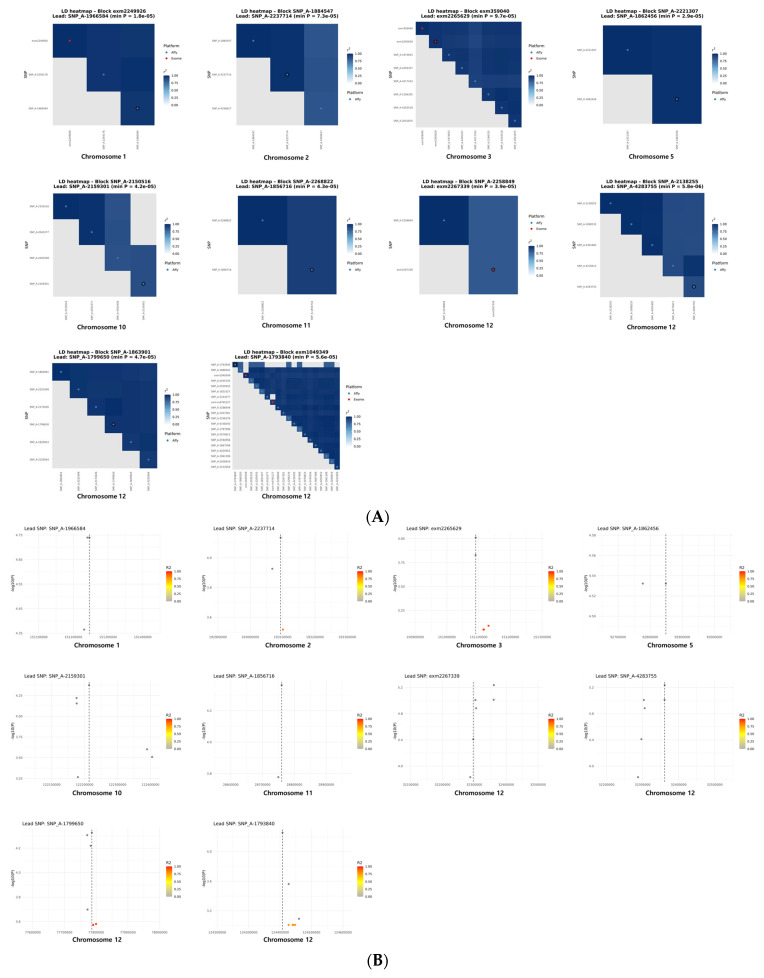
Cross-platform LD block architecture and regional association patterns for SNPs. (**A**) LD heatmaps for each block containing a SNP with *p* < 1 × 10^−4^. SNPs are ordered by genomic position, colored by platform (Affymetrix Axiom Exome Array: blue; Illumina Infinium HumanExome BeadChip: red), with lead SNPs outlined. These heatmaps summarize local LD patterns and cross-platform concordance. (**B**) Regional association plots showing −log_10_(*p*) values within ±150 kb of each lead SNP. Points are colored by LD (r^2^) to illustrate LD decay, and the vertical dashed line marks the lead SNP. These plots highlight the peak signal and its surrounding LD structure. In the LD heatmaps, the circled marker indicates the lead SNP. In the regional association plots, each point represents a SNP and the vertical dashed line indicates the genomic position of the lead SNP.

Across the genome, we found ten LD blocks that included at least one SNP with *p* < 1 × 10^−4^. These blocks were spread across chromosomes 1, 2, 3, 5, 10, 11, and 12. Their structures were not uniform: some blocks consisted of only two SNPs, whereas others formed broader clusters containing more than 20 correlated variants. The list of SNPs included in each LD block is provided in Table A1. Chromosome 12 was particularly notable, as it contained four independent blocks corresponding to the *NAV2*, *BICD1*, *CCDC92*, and *ZNF664* loci. To characterize these blocks in more detail, we visualized pairwise LD patterns using cross-platform LD heatmaps (Figure 5A). These heatmaps highlight how SNPs from the Illumina and Affymetrix exome arrays clustered together within each region, revealing both simple and complex LD structures depending on the locus. In parallel, regional association plots (Figure 5B) were generated to show the distribution of −log_10_(*p*) values surrounding each lead SNP, allowing us to examine the strength and breadth of association signals within each block.

## 4. Discussion

This study examined the genetic influence on the development of osteoporosis risk and low BMD in postmenopausal women. Table 1 presents the characteristics of the study subjects. Although the age at menopause was similar between groups, the prevalence of osteoporosis may increase with advancing age after menopause as with a previous study [33]. Underweight is generally associated with lower BMD and a higher risk of fracture; therefore, underweight individuals were excluded from this study [34]. Interestingly, the low BMD group showed higher body weight and BMI, which is contradictory to the general trend. However, both groups were classified as obese according to BMI criteria [35]. This finding may be related to the observation that DR-SOS, DR-T, and DR-Z did not differ significantly between groups, whereas MZ-SOS, MZ-T, and MZ-Z did. The radius is less influenced by walking or daily loading, whereas the tibia supports body weight, making bone loss in the tibia more pronounced. This functional difference may partly explain the variations in body weight and BMI [36]. Overall, postmenopausal women tend to show marked bone loss in the cortical midshaft tibia, whereas differences in the cancellous distal radius are relatively minimal or statistically insignificant.

Logistic regression analysis revealed that no SNPs met the genome-wide significance threshold [37]. However, one SNP exceeded the suggestive significance threshold (*p* < 1 × 10^−5^) on the Illumina Infinium HumanExome BeadChip. This SNP, rs2076212 of the *PNPLA3* gene, is located within the coding region and represents a missense mutation (G115C). It was the only SNP to show statistical significance with an FDR of 0.016 (*p* < 0.05). *PNPLA3* is a gene related to metabolism [38], and a previous study reported its association with osteoporosis [39]. That study also utilized KoGES data but included men, whereas the present study targeted only postmenopausal women. Notably, *PNPLA3* is absent from the Affymetrix Axiom Exome Array, meaning it would not have been detected using Affymetrix alone. This highlights the advantage of expanded analysis using both platforms.

A comparatively larger number of overlapping SNPs and genes were observed on chromosomes 1, 3, 6, 9, 11, and 12. Many of these genes are involved in immune response (e.g., *HLA*-*DOA*) [40], cell signaling (e.g., *DISC1*, *FGF12*) [41,42], and bone metabolism (e.g., *COL6A5*) [43].

LDA revealed that certain genes, such as *ATAD5*, *DNHD1*, *TNFSF15*, and *CCDC92*, were recurrent across both platforms, suggesting that these variants may contribute to the discrimination between disease and control groups. In contrast, random forest and XGBoost model showed relatively low rates of common SNPs across platforms, and genes not identified by LDA appeared at the top of their rankings. This discrepancy likely reflects the fact that LDA selects variables contributing to classification based on linear discriminant criteria, whereas random forest and XGBoost consider nonlinear interactions. These findings indicate that the application of diverse models can be beneficial, since linear and non-linear methods may identify independent subsets of variants. Variants consistently detected across models are thus likely to represent particularly robust candidates [44]. SNPs that recur across platforms are more likely to be associated with disease and, therefore, should be prioritized in further analysis. Applying multiple models in parallel to identify overlapping variant candidates can provide more robust and reliable results [45].

To further narrow down the variants with potential biological relevance, we incorporated multiple in silico prediction tools. This approach allowed us to focus on SNPs that consistently showed signs of functional impact rather than relying on any single algorithm. Among the 110 candidates, six variants stood out by receiving deleterious predictions from several independent models, suggesting that they may meaningfully influence bone-related pathways. In particular, *CCDC92* p.Ser70Cys and *DYNC2H1* p.Arg2871Pro demonstrated strong and concordant signals, including high CADD phred scores (>20), which strengthens the likelihood that these variants play a functional role. Taken together, these findings present how integrating diverse computational assessments can help highlight variants that deserve closer biological or mechanistic investigation.

Conservation analysis provided additional support for prioritizing variants with potential functional significance. Variants with high GERP++, phyloP, and phastCons scores—particularly those in *DYNC2H1* and *NQO1*—showed strong evolutionary constraint, reinforcing the pathogenic signals suggested by other in silico predictions. Among the prioritized SNPs, *ZNF510* (rs2289651), *ARMS2* (rs10490924), *DYNC2H1* (rs589623), *CCDC92* (rs11057401), *PTPRB* (rs2584021), and *NQO1* (rs1800566) were consistently predicted to have deleterious effects. Several of these variants also ranked highly in our machine-learning models: *CCDC92* in LDA, *DYNC2H1* in XGBoost, and *PTPRB* in random forest. Together, these convergent lines of evidence suggest that these variants may contribute meaningfully to osteoporosis susceptibility in our cohort. In contrast, variants with weak statistical associations or low conservation scores may act through mechanisms less tied to evolutionary constraint or may reflect population-specific genetic variation.

Although the overall PPI enrichment was not statistically significant (PPI enrichment *p* > 0.05), a small cytoskeleton-related cluster could still be observed. Cluster analysis revealed an over-representation of genes related to cytoskeletal organization and cell projections, which may reflect the importance of mechanosensing and cell architecture regulation in bone remodeling. In particular, significant enrichment in the cytoplasmic region of membrane-bound cell projections and distal axon terminals suggests that osteogenic regulation may be mediated through pathways related to microtubule dynamics and cellular projection processes [46,47,48].

In this study, we investigated the genetic determinants of osteoporosis risk and low BMD in postmenopausal women using two complementary genotyping platforms and a multilayered analytic approach that included statistical testing, machine learning, in silico functional prediction, and protein–protein interaction analysis. Although genome-wide significance was not achieved, the suggestive association of rs2076212 (*PNPLA3*) highlights the benefit of integrating two platforms, as this variant was present only on the Illumina array and would not have been captured by a single-platform analysis.

Although *PNPLA3* was the only variant to exceed the suggestive significance threshold (*p* < 1 × 10^−5^) in the single-SNP analyses across both platforms, integrating the datasets at the LD-block level revealed several additional loci with similarly strong association patterns. Because the Illumina and Affymetrix arrays differ substantially in variant coverage, many variants present on one platform are entirely absent from the other, making single-SNP testing inherently incomplete. By grouping correlated variants into LD-defined genomic regions using SNPs from both arrays, we were able to recover shared genetic signals that were not detectable from individual *p*-values alone. This LD-based approach noticeably improved the sensitivity of our analysis and helped reveal additional loci with potential biological relevance. In particular, four distinct regions on chromosome 12—*NAV2*, *BICD1*, *CCDC92*, and *ZNF664*—became apparent only when LD patterns were examined collectively across the two platforms, even though no individual SNP within these regions met strict significance thresholds on its own. These observations illustrate how LD-block integration can bridge platform-specific gaps, reduce discrepancies between arrays, and uncover extra genetic signals that may play a role in osteoporosis susceptibility.

A number of overlapping SNPs were repeatedly detected across chromosomes enriched in genes related to immune regulation, cell signaling, and bone metabolism. Among the machine-learning models, LDA showed greater cross-platform concordance than random forest and XGBoost model, suggesting that SNPs selected by multiple models and multiple platforms represent high-confidence candidates. Six variants (*ARMS2*, *CCDC92*, *NQO1*, *ZNF510*, *PTPRB*, and *DYNC2H1*) were consistently predicted to be functionally deleterious by ≥3 in silico tools, of which *CCDC92* p.Ser70Cys and *DYNC2H1* p.Arg2871Pro also showed high CADD scores and strong contributions in the machine-learning models. Furthermore, conservation scores supported the potential pathogenicity of *DYNC2H1* and *NQO1*, reinforcing the likelihood of their involvement in osteoporosis risk. Interestingly, *CCDC92* is known to be associated with adipose tissue biology and metabolic traits, whereas *DYNC2H1* encodes a motor protein involved in intracellular transport and cytoskeletal regulation, suggesting that these genes may influence bone remodeling indirectly through mechanosensing and metabolic pathways [49,50,51]. In addition, *NQO1* p.Pro187Ser, a variant involved in the oxidative stress response, may affect bone homeostasis through redox-mediated mechanisms [52]. *PTPRB* encodes a receptor-type tyrosine phosphatase that regulates angiogenesis and vascular integrity by modulating *VEGFR2* signaling in endothelial cells [53]. These functional interpretations are consistent with our STRING clustering results, which revealed enrichment of cytoskeleton- and membrane-projection–related processes, indicating that mechanosensing and cell architecture could be key mediators of bone loss in postmenopausal women. In addition to the model-based results, the LD-block analysis offered an independent layer of evidence for several of these regions. Genes such as *NAV2*, *BICD1*, *CCDC92*, and *ZNF664* appeared as clearly defined LD loci, even though none of the individual SNPs within these regions met genome-wide significance on their own. Notably, several genes highlighted by the machine-learning and in silico analyses—such as *CCDC92*, *NQO1*, and *DYNC2H1*—were also situated within these LD-structured regions. This overlap indicates that different analytic approaches were pointing to the same genomic areas, which strengthens confidence in their biological relevance. By grouping correlated variants into shared LD blocks, this approach also captured regional signals that would have been missed by single-SNP testing alone, allowing variants with modest *p*-values to be interpreted within a broader genetic context [54,55]. Together, these findings show how LD-block integration complements model-based prioritization and helps reveal additional loci that may contribute to osteoporosis risk.

Although this study was exploratory in nature, the identified candidate genes may have several potential clinical implications. First, when integrated with clinical and demographic factors, these genes may contribute to genetic risk assessment and stratification for low bone mineral density in postmenopausal women. Second, the associated genes are involved in biological pathways related to bone metabolism, mechanosensing, metabolic regulation, and cytoskeletal organization, making them promising candidates for targeted functional studies. Finally, the findings of this study may serve as a basis for hypothesis generation in future research aimed at evaluating biomarkers or therapeutic targets. However, additional replication studies and experimental validation are required before these findings can be translated into clinical practice or drug development.

This study combined two genotyping platforms and multiple analytic approaches—including machine learning, in silico prediction, conservation scoring, and network analysis—to strengthen the identification of biologically meaningful variants. Nevertheless, several limitations should be acknowledged. The final analytic sample size after quality control was modest, which may have limited statistical power to detect variants with modest effect sizes. Post hoc power analyses showed that many overlapping SNPs exceeded the predefined post hoc power threshold; however, several variants exhibited low post hoc power, and some statistically significant signals were observed despite limited power, indicating that these associations should be interpreted with caution. Overall, the modest sample size limited the study’s ability to reliably detect small-to-moderate genetic effects. This was further supported by minimum detectable effect size analyses, which showed that none of the observed associations exceeded the odds ratio required to achieve 80% power, emphasizing the need for validation in larger cohorts. Our study was conducted exclusively in Korean postmenopausal women. While this design inevitably limits the direct generalizability of the findings to other ancestral groups and does not fully exclude population-specific effects, the relative homogeneity of the cohort also represents a practical strength from a genetic epidemiology perspective. By reducing background genetic and environmental variability, this homogeneity can facilitate clearer detection of genetic signals within a defined population.

To address limitations related to the modest sample size and platform-specific SNP coverage, we integrated results from the two genotyping platforms using an LD-block–based framework. By analyzing correlated variants as regional units rather than relying solely on single-SNP tests, we were able to capture broader association patterns that were not apparent at the individual-variant level, particularly in genomic regions where the two arrays provided complementary, non-overlapping coverage. Notably, several regions identified through the LD-block analysis were concordant with loci highlighted by machine-learning and functional prediction approaches, providing convergent support across independent analytic strategies. Nevertheless, these findings should be regarded as hypothesis-generating. Validation in larger, independent cohorts—ideally incorporating multi-ethnic populations—and the use of higher-resolution genomic data will be essential to confirm the implicated loci and to more comprehensively define the genetic architecture of osteoporosis.

## 5. Conclusions

Taken together, this study highlights the genetic contributors to postmenopausal osteoporosis risk by integrating two genotyping platforms with LD-block analysis, machine learning, and in silico predictions. Although only *PNPLA3* reached suggestive significance in single-SNP testing, LD-based refinement revealed additional loci—including *NAV2*, *BICD1*, *CCDC92*, and *ZNF664*—that were detectable only when cross-platform LD structure was considered. Convergent evidence across analytic layers prioritized variants such as *CCDC92*, *DYNC2H1*, and *NQO1* as biologically meaningful candidates. These findings underscore the value of combining multi-platform data with structural and functional analyses and provide a focused set of variants for future validation.

## Figures and Tables

**Table 1:** Basic characteristics of the postmenopausal women in this study. DR-T/DR-Z and MT-T/MT-Z denote QUS-derived T- and Z-scores from SOS measurements at the distal radius (DR) and midshaft tibia (MT), respectively. These indices were used for the operational definition of osteoporosis risk/low BMD and are not directly comparable to DXA-based WHO/ISCD diagnostic criteria.

	Control	Low BMD	*p*-Value
age (years)	53.99 ± 4.55	58.09 ± 4.24	0.000
age at menopausal (years)	49.59 ± 2.87	49.47 ± 3.8	>0.05
weight (kg)	64.05 ± 5.15	65.54 ± 6.88	0.034
BMI (kg/m^2^)	25.98 ± 2.35	27.57 ± 2.88	0.000
alcohol consumption (g/day)	0.59 ± 1.4	0.57 ± 1.55	>0.05
calcium consumption (mg/day)	<1000	<1000	-
DR-SOS (m/s)	4200.76 ± 118.27	4000.31 ± 177.36	>0.05
DR-T (m/s)	0.3 ± 0.9	−1.29 ± 1.46	>0.05
DR-Z (m/s)	1.12 ± 1.09	0.1 ± 1.39	>0.05
MT-SOS (m/s)	3936.63 ± 61.31	3625.35 ± 122.15	0.000
MT-T (m/s)	−0.2 ± 0.59	−3.24 ± 1.19	0.000
MT-Z (m/s)	0.59 ± 0.73	−1.92 ± 1.32	0.000

**Table 2:** Overlapping SNPs and Genes by Chromosome.

Chr	*n*	Gene
1	8	*KIF1B*, *ZYG11A*, *PROK1*, *DISC1*
2	6	*NRXN1*, *MARCO*, *CWC22*, *MAP2*
3	11	*C3orf77*, *COL6A5*, *ATR*, *CPB1*, *GFM1*, *FGF12*
4	3	*DGKQ*, *SORBS2*
5	6	*PDZD2*, *C7*, *IQGAP2*
6	16	*NRM*, *NOTCH4*, *HLA-DOA*, *BTBD9*, *THEMIS*, *LAMA2*, *SYNE1*
7	2	*ASNS*
8	5	*SLC18A1*, *C8orf86*, *UBXN2B*, *FAM135B*
9	10	*KDM4C*, *ACER2*, *ZNF510*, *TNFSF15*, *CERCAM*, *SETX*
10	4	*KIAA1217*, *NRG3*, *SH3PXD2A*
11	6	*OSBPL5*, *DNHD1*, *MMP13*, *DYNC2H1*
12	12	*CLECL1*, *SLC4A8*, *MYO1A*, *PTPRB*, *C12orf64*, *CCDC63*, *WDR66*, *GPR81*, *CCDC92*, *ZNF664*
13	4	*SLC46A3*
14	3	*OTX2*
15	2	*TNFAIP8L3*, *LINS*
16	3	*TRAP1*, *NQO1*, *ADAMTS18*
17	3	*DLG4*, *ATAD5*
18	0	
19	1	
20	3	*RNF114*
21	1	*URB1*
X	0	
Y	0	

Chr: chromosome; *n*: number.

**Table 3:** Top 10 SNPs identified by LDA, random forest, and XGBoost models for Illumina Infinium HumanExome BeadChip and Affymetrix Axiom Exome Array.

	LDA			Random Forest			XGBoost		
	rsID	Gene	Coefficient	rsID	Gene	Importance	rsID	Gene	Importance
Illumina Infinium HumanExome BeadChip	rs11657270	*ATAD5*	2.61222	rs2584021	*PTPRB*	0.012048	rs11248060	*DGKQ*	0.01788
	rs4263839	*TNFSF15*	0.424226	rs9554742		0.011968	rs8134971	*URB1*	0.017365
	rs4758423	*DNHD1*	0.38457	rs11124754		0.011813	rs1049674	*ASNS*	0.016935
	rs11057401	*CCDC92*	0.378416	rs10109439	*FAM135B*	0.011802	rs6478108	*TNFSF15*	0.016367
	rs3129304	*HLA-DOA*	0.260162	rs557135		0.011747	rs10253361		0.015085
	rs1049674	*ASNS*	−0.254866	rs4406360		0.011317	rs4679621		0.014259
	rs4633449	*DNHD1*	−0.356667	rs4947122		0.011187	rs6556756		0.014067
	rs6478108	*TNFSF15*	−0.433349	rs6556756		0.011181	rs589623	*DYNC2H1*	0.013548
	rs4765127	*ZNF664*	−0.436117	rs1169081	*WDR66*	0.011081	rs10964136	*ACER2*	0.013402
	rs3816780	*ATAD5*	−2.322621	rs10490924		0.011063	rs763318		0.013336
Affymetrix Axiom Exome Array	rs11057401	*CCDC92*	1.099642	rs2008344	*TRAP1*	0.013578	rs10124818		0.021613
	rs11657270	*ATAD5*	0.777047	rs7305599	*SLC4A8*	0.013159	rs2229032	*ATR*	0.019727
	rs4633449	*DNHD1*	0.437324	rs7514102	*PROK1*	0.01289	rs629648	*THEMIS*	0.018916
	rs4263839	*TNFSF15*	0.393153	rs4758540	*OSBPL5*	0.012731	rs4633449	*DNHD1*	0.015453
	rs11247226	*LINS*	0.315041	rs10253361		0.012213	rs353372		0.014661
	rs6478108	*TNFSF15*	−0.459254	rs8134971	*URB1*	0.012191	rs6033098		0.014627
	rs2229032	*ATR*	−0.525691	rs10109439	*FAM135B*	0.011849	rs6795735		0.014511
	rs4758423	*DNHD1*	−0.567479	rs12033321		0.011474	rs763318		0.013953
	rs3816780	*ATAD5*	−0.896848	rs1009850	*CERCAM*	0.011446	rs10253361		0.013829
	rs4765127	*ZNF664*	−1.089067	rs1169081	*WDR66*	0.011368	rs10748869	*NRG3*	0.013601

**Table 4:** Overlapping SNPs predicted to have deleterious functional effects by multiple in silico algorithms.

SNP ID	Chr	Pos	Gene	Amino Acid Change	SIFT		Polyphen2 HDIV		Polyphen2 HVAR		PROVEAN		REVEL	CADD
					Score	Pred	Score	Pred	Score	Pred	Score	Pred	Score	Phred
rs10490924	10	122,454,932	*ARMS2*	p.Ala69Ser	0	D	0.994	D	0.957	D	−2.63	D	0.061	15.87
rs11057401	12	123,942,759	*CCDC92*	p.Ser70Cys	0.005	D	1	D	0.971	D	−2.44	N	0.164	23.3
rs1800566	16	69,711,242	*NQO1*	p.Pro187Ser	0.032	D	0.438	B	0.167	B	−7.39	D	0.366	24
rs2289651	9	96,774,789	*ZNF510*	p.Gln43Pro	0.01	D	0.838	P	0.202	B	−3.65	D	0.128	22.2
rs2584021	12	70,635,953	*PTPRB*	p.Asp57Tyr	0.004	D	0.978	D	0.77	P	−1.12	N	0.214	20.7
rs589623	11	103,211,861	*DYNC2H1*	p.Arg2871Pro	0.015	D	0.991	D	0.964	D	−4.2	D	0.301	27.4

Chr: chromosome; Pos: position; Pred: prediction; Phred: phred-scaled C-score.

**Table 5:** Evolutionary Conservation Scores (GERP++, phyloP, and phastCons) for Overlapping SNPs Identified Across Both Platforms.

SNP ID	Chr	Pos	Gene	GERP++	phyloP (V)	phyloP (M)	phyloP (P)	phastCons (V)	phastCons (M)	phastCons (P)
rs9284879	3	44,243,092	*TOPAZ1*	2.96	1.407	2.166	−0.106	0.928	1	0.975
rs2289651	9	96,774,789	*ZNF510*	1.52	1.944	−2.174	0.665	0.999	0	0.963
rs10490924	10	122,454,932	*ARMS2*	0.998	0.215		0.618	0.006	0.008	0.025
rs589623	11	103,211,861	*DYNC2H1*	5.76	4.414		0.676	1	1	0.997
rs11057401	12	123,942,759	*CCDC92*	3.44	3.005	1.763	0.661	1	1	0.995
rs2584021	12	70,635,953	*PTPRB*	3.92	0.98	0.848	0.599	0.763	0.446	0.947
rs1800566	16	69,711,242	*NQO1*	5.41	9.295	8.644	0.676	1	1	0.997

Chr: chromosome; Pos: position; (V): vertebrate; (M): mammalian; (P): primate.

**Table 6:** Functional clusters of overlapping genes identified by STRING.

Cluster	Gene Count	Primary Description	Protein Names
1	9	Kinesin binding	KIF1B, DLG4, PTPRB, MAP2, SYNE1, DISC1, NRG3, NRXN1, PDZD2
2	4	miscellaneous	SETX, THEMIS, ATR, ATAD5
3	4	miscellaneous	TRAP1, GFM1, KIAA1217, CWC22
4	2	Mixed, incl. Domain of unknown function DUF4537, and CCDC92/74, N-terminal	CCDC92, ZNF664

## Data Availability

The raw genotype data used in this study were obtained from the Korean Genome and Epidemiology Study (KOGES) under a data-use license and cannot be shared publicly due to institutional and ethical restrictions. Summary-level statistical results generated during this study are available from the corresponding author upon reasonable request.

## Abbreviations

The following abbreviations are used in this manuscript:

KoGES	Korean Genome and Epidemiology Study
IRB	Institutional Review Board
SNP	Single Nucleotide Polymorphism
GWAS	Genome-Wide Association Study
LD	Linkage Disequilibrium
MAF	Minor Allele Frequency
HWE	Hardy–Weinberg Equilibrium
QC	Quality Control
LDA	Linear Discriminant Analysis
PLINK	Persistent Linked INtegrated Kit
GERP++	Genomic Evolutionary Rate Profiling
phyloP	Phylogenetic *p*-value (phylogenetic conservation score)
phastCons	Phylogenetic Hidden Markov Model Conservation Score
CADD	Combined Annotation-Dependent Depletion
SIFT	Sorting Intolerant From Tolerant
PROVEAN	Protein Variation Effect Analyzer
REVEL	Rare Exome Variant Ensemble Learner
STRING	Search Tool for the Retrieval of Interacting Genes/Proteins
DAVID	Database for Annotation, Visualization and Integrated Discovery
MCL	Markov Cluster Algorithm
DR-SOS	Distal Radius Speed of Sound
DR-T	Distal Radius T-score
DR-Z	Distal Radius Z-score
MT-SOS	Midshaft Tibia Speed of Sound
MT-T	Midshaft Tibia T-score
MT-Z	Midshaft Tibia Z-score
BMI	Body Mass Index
WHO	World Health Organization
BMD	Bone Mineral Density
Q-Q plots	Quantile-Quantile plots

## Appendix A

**Table A1:** Chromosomal LD Blocks and Their Constituent SNPs Identified Through Cross-Platform Integration.

Chr	Chip ID	rs ID	CHR	Pos	Gene	Function
1	exm2249926	rs55877187	1	151230929	*PSMD4*	Silent
1	SNP_A-2293176	rs11204791	1	151240542		
1	SNP_A-1966584	rs6587562	1	151246241		
2	SNP_A-1884547	rs2356967	2	193068151		
2	SNP_A-2237714	rs2592273	2	193093850		
2	SNP_A-4256617	rs2356971	2	193101110		
3	exm359040	rs3732765	3	151090424	*MED12L, P2RY12*	Missense_R1210Q, Silent
3	exm2265629	rs9859538	3	151090963	*MED12L, P2RY12*	Silent
3	SNP_A-1974833	rs3821663	3	151100677	*MED12L*	
3	SNP_A-4205327	rs10935840	3	151101083	*MED12L*	
3	SNP_A-4217243	rs17204501	3	151114889	*MED12L*	
3	SNP_A-2166335	rs17204508	3	151115204	*MED12L*	
3	SNP_A-4203518	rs4680406	3	151116816	*MED12L*	
3	SNP_A-2041875	rs2276765	3	151131222	*MED12L*	
5	SNP_A-2221307	rs893547	5	92776972		
5	SNP_A-1862456	rs2344386	5	92848652		
10	SNP_A-2150516	rs2148476	10	122175555		
10	SNP_A-2043377	rs2420717	10	122175979		
10	SNP_A-4303428	rs1326663	10	122179526		
10	SNP_A-2159301	rs10886690	10	122213646	*PPAPDC1A*	
11	SNP_A-2268822	rs1914475	11	28749414		
11	SNP_A-1856716	rs10835398	11	28759826		
12	SNP_A-2258849	rs1798255	12	32287259	*BICD1*	
12	exm2267339	rs2608405	12	32296621	*BICD1*	Silent
12	SNP_A-2138255	rs4931615	12	32303400	*BICD1*	
12	SNP_A-1898535	rs4931616	12	32303456	*BICD1*	
12	SNP_A-4301892	rs2630578	12	32305787	*BICD1*	
12	SNP_A-4278412	rs161962	12	32360803	*BICD1*	
12	SNP_A-4283755	rs161961	12	32361233	*BICD1*	
12	SNP_A-1863901	rs4017759	12	77771495	*NAV2*	
12	SNP_A-2225499	rs1880881	12	77772555	*NAV2*	
12	SNP_A-2174026	rs1527063	12	77782433	*NAV2*	
12	SNP_A-1799650	rs4761376	12	77786244	*NAV2*	
12	SNP_A-1829924	rs1465070	12	77790549	*NAV2*	
12	SNP_A-2229564	rs2011194	12	77799416	*NAV2*	
12	SNP_A-1793840	rs11057394	12	124407676	*DNAH10*	
12	SNP_A-1888303	rs11057401	12	124427306	*CCDC92*	
12	exm1049349	rs11057401	12	124427306	*CCDC92*	Missense_S70C
12	SNP_A-2035335	rs4765219	12	124440110	*CCDC92*	
12	SNP_A-2209355	rs7305864	12	124441880	*CCDC92*	
12	SNP_A-1821027	rs6488914	12	124447841	*CCDC92*	
12	SNP_A-2163277	rs4765127	12	124460167	*ZNF664*	
12	exm-rs4765127	rs4765127	12	124460167	*ZNF664*	Silent
12	SNP_A-2288649	rs12311114	12	124460703	*ZNF664*	
12	SNP_A-2267281	rs4765528	12	124462254	*ZNF664*	
12	SNP_A-2296376	rs11057408	12	124464836	*ZNF664*	
12	SNP_A-4238292	rs7978610	12	124468572	*ZNF664*	
12	SNP_A-1787908	rs7311969	12	124470333	*ZNF664*	
12	SNP_A-2079815	rs7311233	12	124475940	*ZNF664*	
12	SNP_A-2194556	rs4765148	12	124478637	*ZNF664*	
12	SNP_A-1867568	rs4765568	12	124479161	*ZNF664*	
12	SNP_A-4204952	rs11057409	12	124479331	*ZNF664*	
12	SNP_A-1961399	rs7975482	12	124481690	*ZNF664*	
12	SNP_A-2058919	rs1187415	12	124491529	*ZNF664*	
12	SNP_A-2222659	rs7307053	12	124494540	*ZNF664*	
